# Development of a Real-Time qPCR Assay for Quantification of Covert Baculovirus Infections in a Major African Crop Pest

**DOI:** 10.3390/insects6030746

**Published:** 2015-08-25

**Authors:** Robert I. Graham, Yamini Tummala, Glenn Rhodes, Jenny S. Cory, Alan Shirras, David Grzywacz, Kenneth Wilson

**Affiliations:** 1 Lancaster Environment Centre, Lancaster University, Lancaster LA1 4YQ, UK; E-Mails: yaminitummala@gmail.com (Y.T.); ken.wilson@lancaster.ac.uk (K.W.); 2 Lake Ecosystems Group, Centre for Ecology and Hydrology, Bailrigg, Lancaster LA1 4AP, UK; E-Mail: glenn@ceh.ac.uk; 3 Department of Biological Sciences, Simon Fraser University, Burnaby, BC V5A 1S6, Canada; E-Mail: jsc21@sfu.ca; 4 Biomedical and Life Sciences, Lancaster University, Lancaster LA1 4YQ, UK; E-Mail: a.shirras@lancaster.ac.uk; 5 Natural Resources Institute, University of Greenwich, Chatham Maritime, Kent ME4 4TB, UK; E-Mail: d.grzywacz@greenwich.ac.uk

**Keywords:** *Spodoptera exempta*, baculovirus, nucleopolyhedrovirus, covert infections, TaqMan real-time qPCR

## Abstract

Many pathogens and parasites are present in host individuals and populations without any obvious signs of disease. This is particularly true for baculoviruses infecting lepidopteran hosts, where studies have shown that covert persistent viral infections are almost ubiquitous in many species. To date, the infection intensity of covert viruses has rarely been quantified. In this study, we investigated the dynamics of a covert baculovirus infection within the lepidopteran crop pest *Spodoptera exempta*. A real-time quantitative polymerase chain reaction (qPCR) procedure using a 5' nuclease hydrolysis (TaqMan) probe was developed for specific detection and quantification of *Spodoptera exempta* nucleopolyhedrovirus (SpexNPV). The qPCR assay indicated that covert baculovirus dynamics varied considerably over the course of the host life-cycle, with infection load peaking in early larval instars and being lowest in adults and final-instar larvae. Adult dissections indicated that, contrary to expectation, viral load aggregation was highest in the head, wings and legs, and lowest in the thorax and abdomen. The data presented here have broad implications relating to our understanding of transmission patterns of baculoviruses and the role of covert infections in host-pathogen dynamics.

## 1. Introduction

Diseases can be an important factor in the population dynamics of insect pests of agricultural importance as well as being useful model systems for exploring host pathogen interactions. Among the most important insect crop pests are the species of Lepidoptera such as *Spodoptera* spp. and *Heliothis/Helicoverpa* spp. which are globally important crop pests whose conventional control is challenging due to their ability to develop resistance to chemical pesticides. The viral insect diseases caused by Baculoviruses, double-stranded DNA virus [1] are among the most studied and have been developed for both classical biological control and as biological pesticides. These viruses are characterised by having the infectious virions embedded in proteinaceous occlusion bodies (OB) adapted for persistence in the environment and classical horizontal transmission between susceptible hosts [2]. With advances in molecular biology, it has become evident that for many insect viral pathogens (including baculoviruses) the ability to cause persistent non-lethal covert infections within their hosts that are capable of being transmitted from parent to offspring is an important characteristic in the ecology of the virus [3,4,5,6,7,8,9,10,11,12,13,14].

Persistent infections are believed to exist in one of two forms: either as a latent infection in which the virus is non-replicating and transcriptional activity is minimal; or as an infection in which the virus is transcriptionally active and replication occurs at a low level, as widely accepted to occur in baculovirus systems studied to date [5,6,7,8]. The precise mechanism by which covert viruses persist in cells of infected insects without causing cell death remains unclear. Studies have shown that when larvae are subjected to periods of stress (such as poor diet, crowding, or the presence of other pathogens), covert infections may be triggered into lethal overt infections that produce OBs for subsequent horizontal transmission (e.g., [4,15]). However, the various protocols for triggering covert viruses have proved inherently unreliable and unrepeatable indicating that our fundamental knowledge of this phenomenon is deficient and that we have much to learn about the dynamics and behaviour of non-persistent infections and their role in the ecology of lepidopteran populations, especially those of economic and agricultural importance.

The larval stage of the African armyworm moth, *Spodoptera exempta* (Lepidoptera: Noctuidae), is a devastating cyclical and migratory crop pest of maize, wheat, sorghum, and other staple crops in a large part of sub-Saharan Africa [16]. High-density outbreaks of armyworm caterpillars typically exceed densities of 100–200 larvae per m^2^. The adult moth is highly migratory, and most armyworm outbreaks are characterized as single-generation eruptions [16]. *S. exempta* harbours a baculovirus exhibiting both overt horizontal [17,18] and covert vertical [8,19,20] transmission strategies. Overt infections of *S. exempta* nucleopolyhedrovirus (SpexNPV) are prevalent in natural populations [17] and SpexNPV has been known to cause up to 98% mortality in some natural populations, though infection levels are highly variable and generally much lower than this [16,21]. While it has been shown that covert infections occur in SpexNPV and it is hypothesised that they play an important role in the seasonal population cycle [8], very little is known about the population dynamics of the covert form of this baculovirus or how the switch from covert to overt infection is initiated. One hypothesis that has been proposed is that covert infections need to reach a certain threshold intensity or viral load before they can be triggered into overt infections. Greater insight into this process could be a key to better understanding of the dynamics of SpexNPV and thus to better prediction of pest outbreaks and to an understanding of how to manipulate the host pathogen system to reduce pest outbreaks and crop damage.

Here, we report on the design and implementation of a real-time qPCR technique allowing the assessment of covert baculovirus infections within both larval and adult forms of the host moth. Specifically, the qPCR assay was used to: (i) quantify the dynamics of covert SpexNPV infections over the course of host development; (ii) determine the principal body parts harbouring covert infections; and (iii) track the replication dynamics of an orally-induced overt infection. Assessing these three parameters allowed us to quantify the infection intensity (viral load) dynamics of asymptomatic covert infections, evaluate hypotheses of covert to overt triggering and thereby significantly furthering our knowledge of host-pathogen interactions within this major agricultural crop pest.

## 2. Experimental Section

### 2.1. Culture of S. exempta

The *Spodoptera exempta* laboratory culture was collected from central Tanzania in January 2011, and maintained on standard culture protocol on a wheatgerm diet [8,22] at 24 °C under a 12 h light/dark cycle. Insects used for experiments were at least two generations under laboratory conditions.

### 2.2. Overt Baculovirus Provenance

*Spodoptera exempta* nucleopolyhedrovirus (SpexNPV) was collected from a single larval cadaver in central Tanzania in 2008 and isolated using standard centrifugation techniques, as previously described [23]. The concentration of SpexNPV occlusion bodies (OBs) of each virus preparation was measured using a Neubauer Improved haemocytometer, with replicated samples taken twice at two dilutions. Viral DNA was extracted as described in Graham *et al.* [24]. Briefly, OBs were lysed by addition of 0.5 M Na_2_CO_3_, 0.1% SDS and incubated for 2 h at 37 °C with proteinase K (200 mg/mL). DNA was purified by phenol/chloroform extraction, dialysed in 1× TE buffer, and stored at 4 °C until required.

### 2.3. Total Genomic DNA Extraction from Insects

All insects used in this study were sourced from the laboratory culture where there was no history of viral contamination or spontaneous outbreaks. However, to remove any potential contaminants from our samples (such as surface pathogens, including NPV OBs), insects used for DNA extraction were surfaced-sterilised using 10% hypochlorite solution, and then washed twice in 70% ethanol. Total genomic DNA was extracted from whole armyworm adults and larvae using the DNA/RNA Allprep Kit (Qiagen Ltd., Crawley, UK), according to the manufacturer’s instructions. DNA quantification was undertaken using a Nanodrop2000 Spectrophotometer (ThermoScientific, Willimington, DE, USA). DNA quality was assessed by testing the 260/280 nm ratio readings during the quantification process, and amplifying the *S. exempta* mitochondrial cytochrome oxidase I (COI) gene, using universal primers LCO-1490 and HCO-1298 [25].

### 2.4. Real-Time Quantitative PCR (qPCR)

Conceptual considerations and nomenclature for real-time qPCR were carried out and reported here in accordance with the MIQE guidelines [26]. Primer Express software (v3.0; Applied Biosystems, Warrington, UK) was used to design primers and the hydrolysis (Taqman, Thermo Fisher Scientific, Warrington, UK) probe specific to the SpexNPV *polyhedrin* gene in the Tanzania isolate (Accession number JX488468). The forward primer (P1) sequence 5'-CCCGTGTACGTAGGAAACAACA-3', reverse primer (P2) 5'-CAACCGCCGCCCTTCT-3' and hydrolysis probe (TaqMan) 5'-6FAM-CGAGTACCGCATCAGCCTGGCC-TAMRA-3' amplified a 62 bp region of the SpexNPV *polyhedrin* gene. To check for target specificity *in silico*, the viral *polyhedrin* sequence and the primer sequences were compared with sequences published on NCBI GenBank database, using the online basic local alignment search tool (BLAST). A perfect match along the entire length of the primers was found only for the *polyhedrin* target gene sequence.

Template DNA (5 µL) was used in 25 µL reactions containing 12.5 µL 2× TaqMan Universal PCR Master Mix (P/N 4304437, Life Technologies, UK) and 0.4 µM each of primers P1 and P2 (P/N 4304972, Life Technologies, UK) and hydrolysis probe (P/N 450003, Life Technologies, UK, UK). Reactions were run on an ABI Prism 7000 SDS machine (Applied Biosystems) in triplicate, using a thermal cycling program consisting of an initial denaturation step at 95 °C for 10 min, followed by 40 cycles of 95 °C for 15 s and 60 °C for 60 s.

For each qPCR assay, a standard curve was constructed using 10-fold serial dilutions of viral genomes, originally isolated from pure NPV OBs (range, 5–5 × 10^6^ viral genomes); and each dilution was processed in triplicate on the same 96-well PCR plate with the samples. SpexNPV genome size was determined from a complete viral-genome sequence (genome size of 129.5 Kbp; Escasa and Cory, unpublished data [27]). Negative controls (“no template controls” NTC; water instead of template DNA) were included in all reactions. Data were analysed using Sequence Detection Software (v1.2.3) 7000 (Applied Biosystems, Warrington, UK) and SpexNPV viral loads were reported as number of viral genomes per µg of total DNA. Only standard curves in which the regression coefficients of determination (R^2^) exceeded 0.990 were considered sufficiently accurate for determination of persistent virus levels. PCR efficiency was calculated using the equation, Efficiency = −1 + 10^(−1/slope)^ [28].

### 2.5. Covert Virus Dynamics during S. exempta Development

Three pairs of 2nd generation laboratory adults were used to establish a time-course experiment to investigate covert-virus dynamics over host development. Mated females laid eggs for 2–3 days and were then frozen for subsequent analysis. Eggs were placed in 30 mL polypots containing artificial diet. When neonates emerged, approximately 30 insects per pair were individually reared in 30 mL diet pots at 24 °C. Four individuals per pair were sacrificed after 4, 6, 8, 10, and 12 days post hatch, and stored at −20 °C in 100% ethanol. Total DNA from insects was extracted as above, and TaqMan qPCR used to investigate SpexNPV load. Head capsule widths were recorded as an additional function of larval age.

### 2.6. Localisation of SpexNPV Infection within Body Regions of Adult Moths

Twenty-four adult moths were randomly selected from our laboratory culture and dissected into five body parts (abdomen, thorax, head, legs, and wings) under a Leica MZ7.5 stereo microscope (Leica, Milton Keynes, UK). Total genomic DNA was isolated from each individual body region, and qPCR undertaken to investigate viral load within specific body parts (groups of three individuals were pooled in order to provide sufficient DNA for quantitation).

### 2.7. Baculovirus Dynamics Following SpexNPV Ingestion

Newly-moulted 4th-instar larvae (L_4_) were allowed to ingest a diet-plug (*n* = 27 larvae per treatment) treated with either distilled H_2_O (control) or 1 × 10^4^ SpexNPV OBs. After 24 h, larvae that had eaten all the diet-plug and virus were transferred to individual polypots (30 mL) containing artificial diet. Larvae were kept at 24 °C and checked every 24 h thereafter. A subset of larvae from both treatment groups was sacrificed at each 24 h time-point to monitor baculovirus dynamics.

## 3. Results

### 3.1. Analytical Sensitivity of the qPCR Assay

Serial 10-fold dilutions of purified SpexNPV DNA were used to produce a qPCR standard curve and determine the cut-off value for the assay. The assay was shown to be reproducible (*r^2^* > 0.990) and amplified *polyhedrin* across a seven log serial dilution range with an efficiency of 99.5% (Figure S1, supplementary material). The most reliable and repeatable limit of detection (LOD) was equivalent to 5 viral genome copies of SpexNPV (approximate Cq of 39).

### 3.2. Covert SpexNPV Dynamics during Host Developmental Cycle

**Figure 1:**
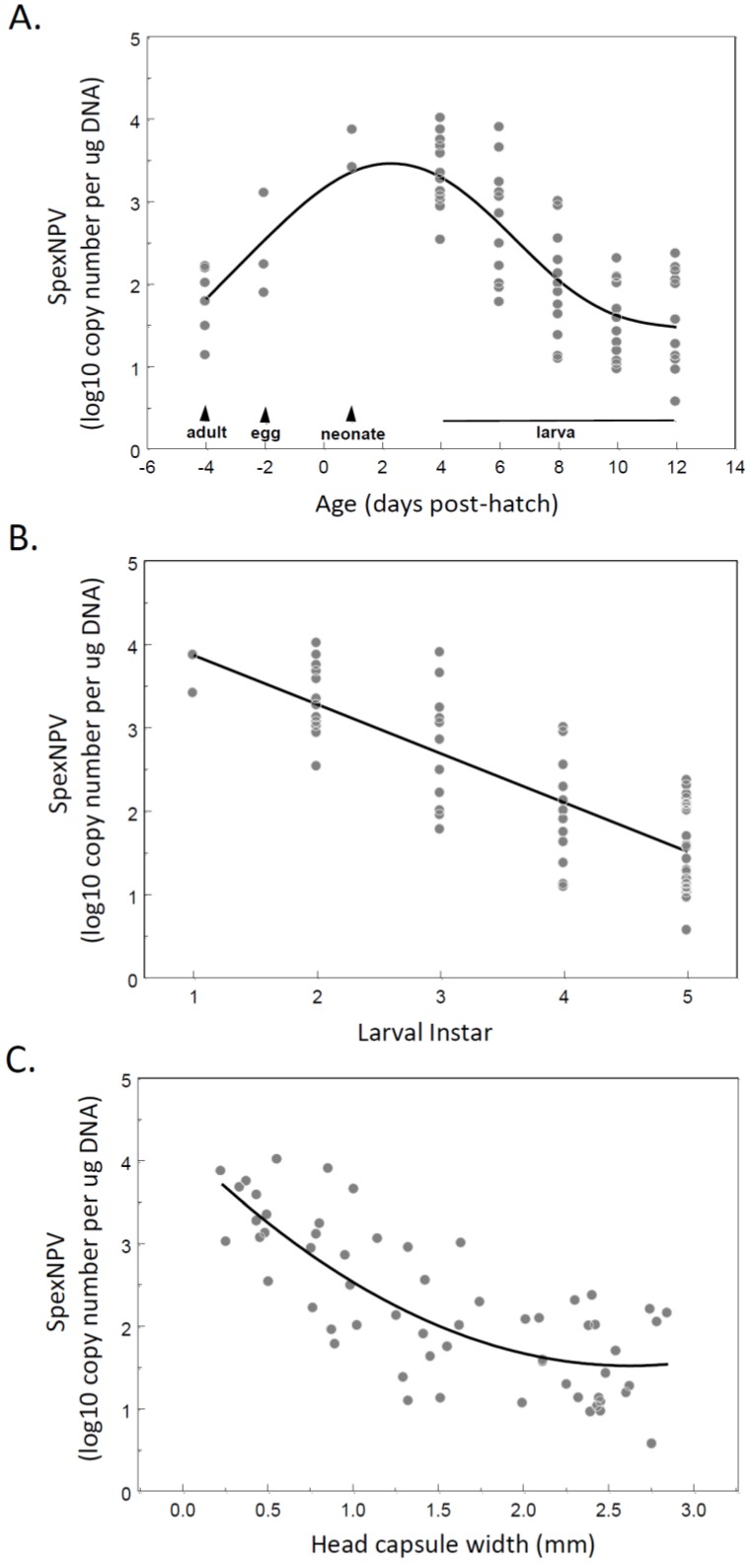
Covert SpexNPV DNA as measured in asymptomatic insects sampled as adults, eggs, neonates, and 4, 6, 8, 10 and 12 days post egg hatch. SpexNPV load as a function of (**A**) age (days post-hatch); (**B**) larval instar (1st to 5th); and (**C**) head capsule width (mm). In (A) the curve shown is the smoothing spline through the raw data. In (B,C) the linear regression lines are shown.

To determine whether SpexNPV viral load changed over the course of host developmental cycle, a time-course experiment was established in which *S. exempta* larvae were sacrificed at specific time-points from the egg stage through to adult maturity (Figure 1a). All insects used in this study were asymptomatic for SpexNPV infection, with no overt viral mortality observed during the experiment. One larva with a very high covert viral load was excluded from the analysis due to undue leverage, however removal of this outlier did not qualitatively alter the results and improved the model fit. Covert viral load was found to vary widely over the course of *S. exempta* development (up to 10^4^-fold). The highest viral copy number was observed at L_2_ and L_3_, with 3349 ± 881 copies/µg DNA and 8180 ± 6953 copies/µg DNA, respectively (*n* = 12, each). Adults (86.5 ± 25.4 copies/µg DNA; *n* = 6) and L_5_ larvae (65.0 ± 13.7 copies/µg DNA; *n* = 24) produced the lowest viral readings. The viral load was higher in egg and neonate stages then there was a decline in viral load as the larva grew post hatching with a linear decline in viral load from 1st instar neonates to 5th instar larvae (Linear regression: viral load = 2.27 – 5.79 × instar; *R*^2^ = 0.64; F_1,59_ = 106.1, *p* < 0.0001; Figure 1b), and a quadratic relationship between viral load and larval head-capsule width (viral load = 2.23 – 5.20 × head-capsule + 1.50 × head-capsule^2^; *R*^2^ = 0.61; F_2,56_ = 43.3, *p* < 0.0001; Figure 1c).

### 3.3. Localisation of Baculovirus in Adult Moths

**Figure 2:**
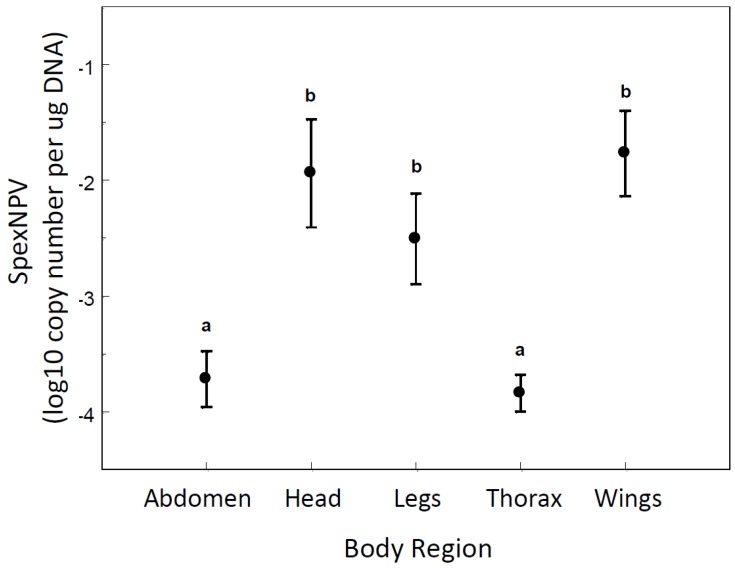
Covert SpexNPV DNA levels in different body regions of adult *S. exempta* moths. Means ± S.E. are shown. Different letters above the error bars indicate means that are significantly different according to Fisher’s least significant difference test.

To determine whether the covert baculovirus was systemic or localised in particular body tissues, adult moths were dissected into five regions (abdomen, thorax, head, legs, and wings). There was a highly significant difference between the body regions in the amount of SpexNPV detected (ANOVA: F_4,34_ = 8.03, *p* = 0.0001; *R*^2^ = 0.49; Figure 2). *Post hoc* Fisher’s least significant difference tests indicated that covert viral loads were significantly higher in the head, legs and wings (log_10_(mean) ± S.E. = −3.780 ± 0.140) than in the abdomen and thorax (−2.084 ± 0.239).

### 3.4. Baculovirus Dynamics Following Oral Ingestion

**Figure 3:**
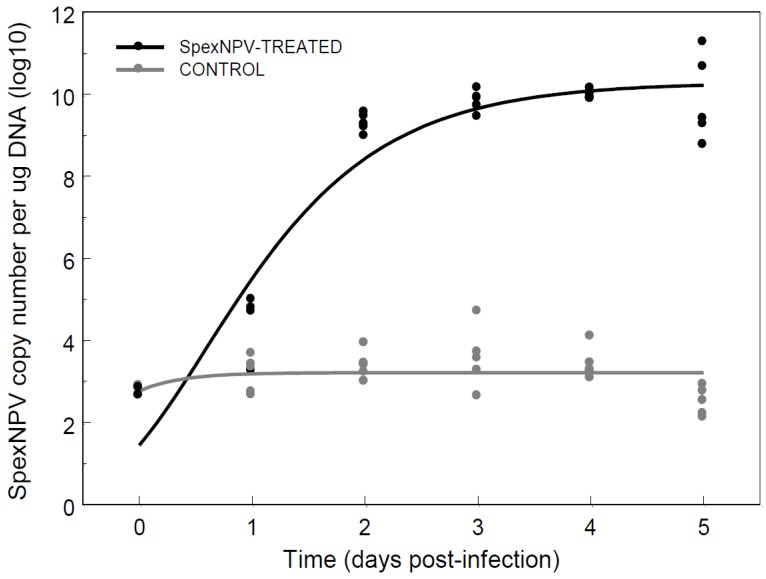
Dynamics of SpexNPV genome replication following ingestion of 10^4^ OBs (treated) and dH_2_O (control). The lines are the fitted Gompertz curves (see text for details).

Using qPCR to track an orally-induced overt infection allowed us to monitor the progress of infection, and give an insight into the dynamics and magnitude of overt baculoviral disease in comparison to covert infections. To quantify the dynamics of viral replication during an overt infection, early L_4_ larvae were fed 1 × 10^4^ SpexNPV OBs. Baculovirus dynamics were monitored throughout the infection process and compared to control larvae that were not inoculated with virus (Figure 3). Initial background covert readings at day 0 (before treatment) were 589 ± 83 (mean ± S.E.) SpexNPV copies/µg DNA. Throughout larval development, control larvae had on average 4.1 ± 1.9 × 10^3^ copies/µg DNA, with viral load being a quadratic function of time post-infection, declining slightly towards the end of the larval instar (Linear regression: Viral load = 3.17 − 0.70 × Time – 2.55 × Time^2^; *R*^2^ = 0.41; F_2,24_ = 8.17, *p* = 0.002). In contrast, in larvae inoculated with SpexNPV viral load increased asymptotically between one and three days post infection to reach an average of 1.3 ± 0.8 × 10^10^ copies/µg DNA (Viral load = 8.27 + 15.32 × Time – 9.01 × Time^2^; *R*^2^ = 0.88; F_2,23_ = 87.35, *p* < 0.0001), with viral load increasing asymptotically with larval age.

Arguably, a better way to describe the SpexNPV dynamics of infected larvae is to fit a Gompertz function to the data: *f*(x) = *a* × exp(–*b* × exp(−*k* × x)). When this non-linear growth function was fitted to the infection data, the parameter estimates (+ S.E.) for *a*, *b* and *k* were as follows: Control larvae: *a* = 3.213 + 0.135, *b* = 0.149 + 0.156, *k* = 2.849 + 0.843; SpexNPV-treated larvae: *a* = 10.286 + 0.325, *b* = 1.963 + 0.371, *k* = 1.144 + 0.193. Only the asymptote parameter, *a*, was significantly different from zero for the model describing SpexNPV dynamics in control larvae, whereas all three model parameters were statistically significant for the dynamics in virus-treated larvae, consistent with viral replication in these insects. All SpexNPV-treated larvae had died by the final time-point, remaining largely asymptomatic until 5 days post infection.

## 4. Discussion

The aim of this study was to produce and validate a reliable qPCR methodology to quantify the dynamics of covert baculovirus infections within the lepidopteran host, *Spodoptera exempta*. To achieve this, we developed a specific real-time qPCR detection assay using a hydrolysis (TaqMan) probe, for fast and successful quantitation of SpexNPV. Covert infections are widespread in insect systems, and baculovirus persistent infections have long been thought to be prevalent in lepidopterans (for example, [17]). Their existence has been used to explain the widely observed phenomena of the appearance of occurrence of overt infection in healthy populations [29] and the finding of heterologous viral progeny after oral-infection with baculoviruses [4,30,31,32,33,34]. Traditional methods for NPV detection include phase-contrast microscopy, homogenization and staining of insect tissues. This requires specialized skills, is not very sensitive or species specific, and can only be used to detect virus in samples with fairly high viral loads, such as infected larvae. Molecular methods now present an opportunity to develop much more sensitive tools for virus detection, such as this new qPCR method.

In our host-development study, covert viral load appeared to peak in early instar larvae (0, 4 and 6 days post hatch), and then decrease markedly with larval age, specifically after 8 days post hatch, by which time the larvae had entered larval-stage L_4_. In an assessment of the closely related *Spodoptera exigua* host-NPV system, Murillo *et al.* [9] used a SYBRgreen qPCR assay. They reported high covert viral loads at L_1_ and L_3_, with lower viral levels at later instars, pupae and adults, concluding that the host may be suppressing infection during these later life-stages. It should be noted that in our study we have looked at “number of viral genomes per µg DNA” whereas Murillo *et al.* [9] looked at pg DNA. This should be noted when comparing different published studies. In common with many other *Spodoptera* species, *S. exempta* is a phase-polyphenic species [22], and it is at L_4_ stage that the cuticular colour differentiation becomes most obvious. It is possible that this cuticular colour change (from green to a black melanic form) and the accumulation of melanin and other immune-associated factors [35,36] could be driving the reduction in viral loads; thus density-dependent prophylaxis (DDP) may directly impact upon covert virus dynamics, with dark melanic forms of larvae having lower viral loads. Indeed, Vilaplana *et al.* [20] showed that in larva from parents reared under solitary conditions, and so which did not show DDP, the viral infection was more prevalent than those from parents reared gregariously. This is consistent with a finding in the field, where covert viral loads were lower in populations of very high larval density [21]. The insects in our current study were reared individually (and therefore DDP *per se* would not be a factor), but individual cuticular melanisation remains a consideration.

When *S. exempta* larvae were subjected to a superinfection via ingestion of SpexNPV OBs, rapid replication of SpexNPV occurred within 24–48 h post infection (the number of virus genomes increased approximately 10^6^-fold). This indicated rapid virus replication, despite larvae remaining largely asymptomatic for a further 72 h. At this stage of our investigations, it is not clear whether the viral replication was due to the ingested virus only, replication of covert virus only, or a combination of the two. It is possible that the covert NPV infection was triggered into rapid replication somehow by the challenge of ingested NPV [4,30,31,32,33,34]. Further investigation whereby *S. exempta* larvae are fed heterologous virus (one that is not detected by this qPCR assay) could resolve the nature of covert virus dynamics when challenged by a competing pathogen.

Vilaplana *et al.* [8] demonstrated that persistent infections of *Spex*NPV were common in *S. exempta* field populations of larvae collected from a small geographical area in northern Tanzania. The detection of virus in adult moths is considered particularly important due to the fact that adults cannot be directly infected by baculoviruses, nor are they thought to support extensive (if any) viral replication [37]. Therefore, detection here of *Spex*NPV in the adult life stage validates a key assumption of the hypothesis of vertical transmission of the virus. Interestingly, our results on the location of virus in covertly infected adult body parts are perhaps contrary to what one might expect: the virus was found in all the body parts tested and not restricted to specific locations or organs. In a viral species adapted to vertical transmission, one would expect high viral loads within the reproductive organs (found in the host abdomen) but in this study, the highest loads were found not in the abdomen but in the wings, head and legs. As yet, we are unsure why this may be, but possibly it is due to virus accumulation in areas of high chitin concentration, or areas of lower haemolymph levels (resulting in the virus being exposed to a reduced immune challenge). However, at this stage we cannot rule out the possibility that the differences are due to an artefact of DNA isolation, with perhaps different body parts having more PCR inhibitory chemicals for which we did not test, although the protocol was undertaken to ensure these would be kept to a minimum. In future experiments, diluting samples 10-, 100- or even 1000-fold before qPCR could enable us to test for possible contaminants. In addition, despite the specificity and sensitivity of our SpexNPV *polyhedrin* primers, we cannot completely rule out the possibility that highly similar and not yet discovered viruses also could be recognized. As discussed by Krokene *et al.* [38], targeting additional genes in the virus genome of interest would make the qPCR method more robust and reduce the chance of detecting highly similar viruses.

In a wider context, baculoviruses present an interesting model system to study due to the presence of what appears to be two transmission strategies (horizontally transmitted, highly pathogenic; and vertically transmitted, seemingly asymptomatic). In the armyworm system, it may be the specific dynamics of the host populations that selects for the occurrence of these two different viral transmission strategies. Armyworm populations alternate during the year between many outbreaks with high populations of gregarious armyworms in the rainy seasons when food is abundant and very low density solitary populations in the dry season when food is scarce. Persistent covert asymptomatic virus strains are most likely the superior competitors (as compared to the highly pathogenic horizontally-transmitted variants) during the dry season (approximately 6 months of the year in Tanzania) when host population densities are extremely low and dispersed [39]. However, when conditions change and high density host populations appear and become suitable for horizontal transmission of the virus, a persistent virus that can reactivate into an overt-infection will become most competitive. To date, modelling approaches suggest that persistent infections could create a variety of host dynamics: from host-pathogen cycles to endemic persistent interactions, whereby the healthy individuals are lost from the host system [40,41,42,43]. The high prevalence of covert virus infection found in field populations of *S. exempta* (>97%, [21]) suggests that the populations studied to date are close to such an endemic fixation in which virus-free insects have been lost.

At present it is not known how long covert infections can persist in a population, although assays on field-collected *S. exempta* show that they can persist for at least seven generations maintaining 100% infection prevalence [8]. Thus we might expect baculovirus variants that produce covert infections to be adapted in some way to vertical transmission, such as by showing reduced pathogenicity. There is some recent evidence from *Spodoptera exigua* NPV that supports this supposition [44], and that vertical-adapted variants may be produced (*i.e.*, variants that only undertake covert transmission). To date we cannot be sure of the precise nature of the covert infection in *S. exempta*, other than to note that the 62 bp amplified region detected by this qPCR assay is identical to overt-SpexNPV sequence data (Genbank accession number JX488468), and differs over the same region from *polyhedrin* in *Spodoptera frugiperda* at only 2 positions (see accession number KC845532). More research on the genetics of covert infections is required to determine the relationship of covert and overt variants, as the qPCR amplicon is too short to attain any meaningful phylogenetic data. However, a previous study investigating SpexNPV covert infections using the *lef-8* gene sequence, Vilaplana *et al.* [8] did observe minor nucleotide variation between covert and overt infections. Thus, it is possible that covert infections of SpexNPV may be restricted to a few closely related variants; or several very different genotypes, as observed in overt SpexNPV populations [18]. Elucidating this issue would help us to understand the potential for wider use of the current qPCR assay or modifications of it for allelic discrimination assays or the use of high resolution melting (HRM) analysis.

## 5. Conclusions

In conclusion, we present a qPCR assay that allows the investigation of covert baculovirus dynamics within a lepidopteran host, in this case the African armyworm, *S. exempta*. From an applied perspective, the existence of covert and sub-lethal infections in pest populations may open up interesting new options for the implementation of biological pest control programs, given that covert infection can be transmitted to their offspring, which in turn may be more likely to succumb to patent NPV disease following application of viral biopesticide [20]. As a result, the role of covert infections could be an important consideration when managing biological pest control programs.

## Supplementary Files

Supplementary File 1
