# Supplementary material for: Development of a Real-Time qPCR Assay for Quantification of Covert Baculovirus Infections in a Major African Crop Pest
Source: Insects. 2015 Aug 25;6(3):746–59. doi: 10.3390/insects6030746 (PMC4598664; doi:10.3390/insects6030746)
Supplement: Supplementary File 1 [file insects-06-00746-s001.docx]

**Supplementary Materials**


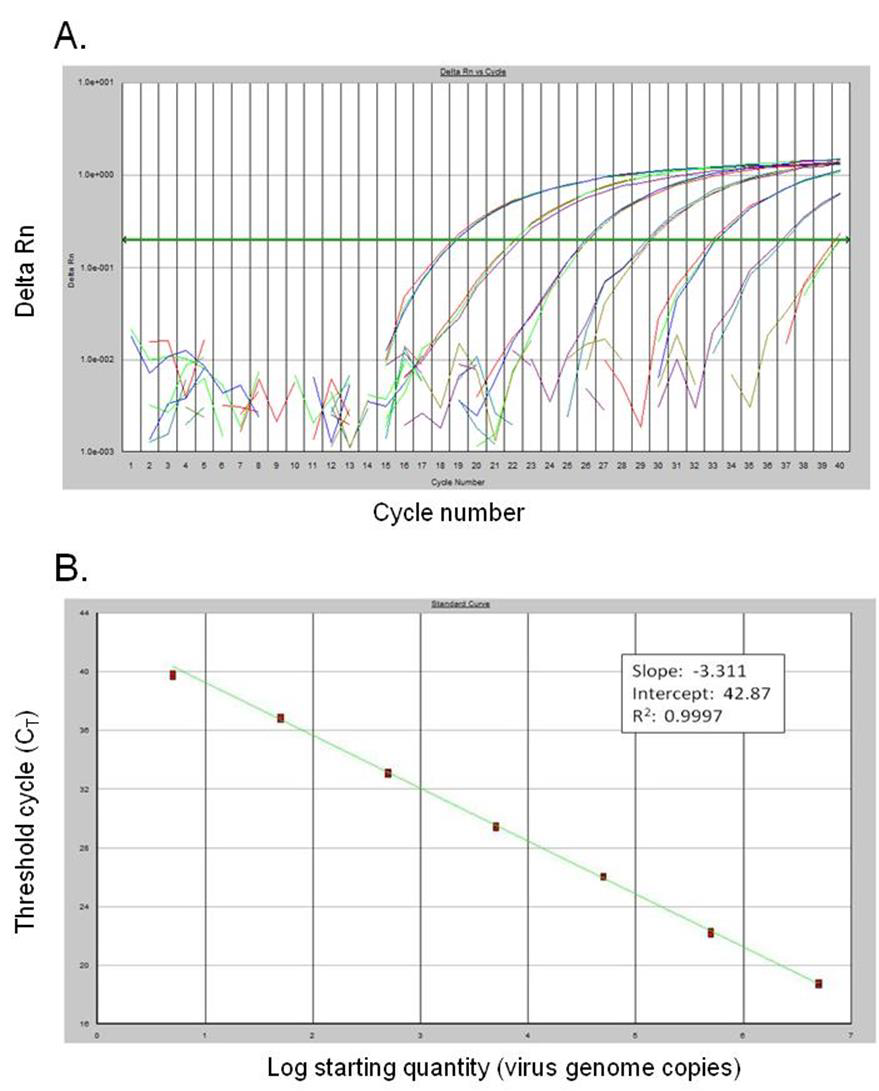


**Figure S1.** Quantitative PCR calibration curve for SpexNPV serial dilutions, within the range of 5 to 5 × 106 viral genomes. (**A**) Quantification curve; (**B**) Standard curve.

© 2015 by the authors; licensee MDPI, Basel, Switzerland. This article is an open access article distributed under the terms and conditions of the Creative Commons Attribution license (http://creativecommons.org/licenses/by/4.0/).
